# Acute Influenza Encephalitis/Encephalopathy Associated with Influenza A in an Incompetent Adult

**DOI:** 10.1155/2020/6616805

**Published:** 2020-12-22

**Authors:** Angela Edet, Katherine Ku, Irene Guzman, Hanadi Abou Dargham

**Affiliations:** ^1^St. Joseph Medical Center, 1800 N California Street, Stockton, California 95204, USA; ^2^Touro University College of Osteopathic Medicine, 1310 Club Drive, Vallejo, California 94592, USA

## Abstract

A 32-year-old male presented to the emergency department (ED) with a productive cough for 4 days and confusion for 2 days prior to presentation. He was febrile, tachycardic, and hypotensive. Initially, labs and influenza A/B PCR were performed. An elevated WBC of 17.3 and a lactate level of 3.1 were noted. He was given a bolus of normal saline and broad spectrum antibiotics, ceftriaxone and azithromycin. The patient was then subsequently found to be positive for influenza A via rapid antigen testing of the nares. On hospital day 2, the patient's mental status and respiratory distress worsened requiring intubation with mechanical ventilation. CT of the head without contrast revealed symmetric areas of hypoattenuation in the frontoparietal deep white matter. Lumbar puncture showed a slight elevation in WBCs and mild lymphocytic pleocytosis. Brain MRI without contrast revealed symmetric hyperintense T2 FLAIR signaling in the periventricular white matter and the splenium of the corpus callosum. He was found to have encephalitis secondary to influenza A and was started on a course of oseltamivir at higher doses of 150 mg BID for 2 weeks. On hospital day 10, after nine days of intubation, the patient received a tracheostomy due to failure to extubate and no improvement in mental status. He remained ventilator-dependent with little improvement in mental status; the patient was transferred to a long-term acute care hospital (LTACH) facility for further specialized care. He did not show any neurologic recovery or improvement in the three months after initial presentation of symptoms. In the fifth month after the initial symptoms, there was no recovery of preinsult neurologic function. The family had a palliative care meeting to discuss the plan and goals of care. It was decided by close family members that “compassionate extubation” would be done due to ongoing stress on the patient's body physically and neurologically. This case illustrates the importance of prompt identification and treatment of influenza in the prevention of rapidly progressive sequelae.

## 1. Introduction

Influenza is the most common cause of infection of the upper respiratory tract during the winter season (November-February) [1]. The infection is usually self-limiting, although children, elderly, immunocompromised, and pregnant women may experience complications [2]. Central nervous system (CNS) involvement is rare with approximately three quarters affecting children, and even less affecting adults [2]. The results of influenza-associated acute encephalopathy (IAE) have a high frequency of neurologic sequelae and death. Altered consciousness, disorientation, and seizures occur within a few days from the onset of fever and respiratory symptoms. In some, symptoms progress to necrotizing encephalitis, deep coma, and death [3].

The influenza virus is uncommonly neuroinvasive, although there have been a small number of published cases described with detection of influenza virus RNA in the cerebrospinal fluid (CSF) via molecular methods [3]. IAE may include a variety of clinical and radiological syndromes frequently associated with viral infections, such as influenza viruses [4]. Cases of IAE have been reported since the 1990s in the Asian continent, especially in Japan. During the 2009 H1N1 pandemic, IAE cases were frequently reported worldwide [1]. Antiviral agents such as neuraminidase inhibitors and immunomodulatory treatments (corticosteroids, intravenous immunoglobulin) are currently administered, but the evidence on their efficacy is limited [2]. Overall, 79% of adults with IAE have survived [1]. 25% of those patients showed lingering neurological deficits, specifically cognitive defects and motor paresis within three months of symptom onset of viral illness [1]. Improved recognition of influenza-associated complications will improve the understanding of the influenza viruses worldwide and allow for better organ-specific supportive care to reduce influenza-associated morbidity and mortality [1].

At the moment, there are no available biomarkers to predict outcome. Available literature on the neurological manifestations of influenza in adults is limited to only single case reports or small case series.

## 2. Case Description

A 32-year-old male with a past medical history of diabetes mellitus (DM) type II and developmental delay presented to the emergency department (ED) with chills, a productive cough, and vomiting for 4 to 5 days and confusion for 2 to 3 days prior to presentation. The patient's baseline prior to respiratory and neurologic symptoms, according to the family, was appropriate. Usually, his demeanor was very outgoing and talkative.

Though he had developmental delay, he was able to hold a job where he took care of developmentally delayed children. He had been in a previous long-term relationship that produced two children for whom he was one of the caregivers. With his history of developmental delay, he was able to follow simple commands. In the ED, the patient was febrile (38.6°C), hypotensive at 93/63 mmHg, and tachycardic at 143 beats/min. On physical examination, the only significant findings were for tachycardia and warmth noted on all four extremities.

A sepsis alert was initiated due to the patient being febrile and tachycardic on presentation. A complete blood count (CBC), complete metabolic panel (CMP), urinalysis (UA), blood culture, lactate level, and influenza A/B PCR were performed. A portable chest X-ray showed diffuse interstitial markings resembling possible pneumonia. The patient was treated with a 2-liter bolus of normal saline, ceftriaxone 1000 mg, and azithromycin 500 mg given once. Ketorolac 15 mg and acetaminophen 1000 mg were given intravenously for pain management.

Laboratory values in the ED were significant for a white blood cell (WBC) count of 17,300/*μ*L (reference level 3,500-9,500/*μ*L); hemoglobin level 11.9 g/dL (13.0-16.5 g/dL); platelet count, 33.4 × 104/*μ*L (15.0 − 35.0 × 104/*μ*L); international normalized ratio of prothrombin time, 1.2 (0.85-1.13); activated partial thromboplastin time, 23.9 seconds (26.1-35.8 s); aspartate aminotransferase, 65 Int Unit/L (1-35 U/L); alanine aminotransferase, 36 U/L (7-42 U/L); total bilirubin, 0.9 mg/dL (0.3-1.2 mg/dL); total protein, 7.8 g/dL (6.5-8.0 g/dL); albumin, 3.8 g/dL (3.8-4.9 g/dL); creatinine, 4.3 mg/dL (0.6-1.1 mg/dL); blood urea nitrogen, 63 mg/dL (8-22 mg/dL); blood glucose level, 111 mg/dL; hemoglobin A1c,13.9% (<6.2%).

The differential diagnosis includes but is not limited to pneumonia, upper respiratory infection, reactive airway disease, and bacterial or viral encephalitis/encephalopathy.

An influenza A rapid antigen test of the nares was positive, and the patient was treated with the antiviral drug oseltamivir 75 mg once in the ED initially. On hospital day 2, though the patient did not manifest convulsions, his state of consciousness deteriorated and he showed a progressive nature of acute respiratory failure. He was intubated for airway protection due to altered mental status and worsening respiratory distress requiring mechanical ventilator support. He was then transferred to critical care for specialized care. A chest X-ray at that time showed right lower lobe consolidation signifying pneumonia.

Initial blood cultures were negative showing no growth in five days. Fungal and viral serology was ordered. Cryptococcus antigen, coccidiodes antibodies IgM, IgG, herpes simplex virus HSV1/HSV2 antigen, and West Nile serology were negative. Aspergillus antigen was also negative.

A lumbar puncture was then performed which showed clear CSF. Gram stain, fungal, and acid fast bacilli culture of CSF was negative. Cultures from spinal fluid showed normal glucose (52 mg/dL) and protein (44 mg/dL). CSF IgG was slightly elevated at 3.7 mg/dL. WBCs were slightly elevated (9 cells/*μ*L) signifying mild lymphocytic pleocytosis.

Infectious disease was consulted in regard to the positive influenza A results. Due to patient's acute renal failure, his oseltamivir was renally dosed until his kidney function showed some improvement. After the initial dose of 75 mg of oseltamivir in the ED, the dose was decreased to 30 mg daily then increased to 75 mg twice daily for approximately five days. After his kidney function mildly improved, the dosing was increased to 150 mg twice daily for two weeks.

An electroencephalogram (EEG) finding showed a diffuse pattern with more irregularity of cerebral function in the frontotemporal head regions. Delta activity suggested some disturbance of deep midline structures. A metabolic, toxic, infectious, or even a disseminated encephalomyelitis etiology could be considered.

On day 4, a computed tomography (CT) of the head without contrast was ordered due to the ongoing alteration in the patient's mental status. The gray-white junction was intact. Periventricular areas showed low density. No hemorrhaging, mass effect, or extra-axial collection was noted. Ventricles and sulci were normal in size and configuration for the patient's age. Later in the evening, the patient was noted to have abnormal decerebrate posturing. Another CT of the head without contrast revealed increased gray-white differentiation for patient's age. Symmetric areas of hypoattenuation in the frontoparietal deep white matter bilaterally, paranasal sinusitis, and mastoiditis.

**Figure 1:**
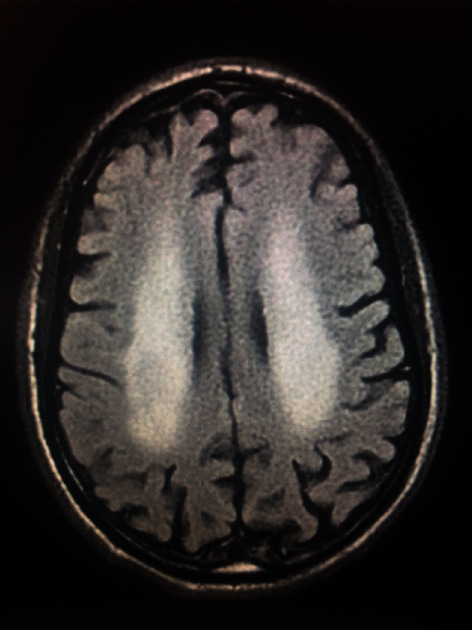
Brain MRI without contrast revealed symmetric hyperintense T2 FLAIR signaling in the periventricular white matter and the splenium of the corpus callosum.

The patient was weaned off sedatives and continued to have encephalopathy. Brain magnetic resonance imaging (MRI) without contrast revealed symmetric high T2 FLAIR signal involving the periventricular white matter and splenium of the corpus callosum with associated restricted diffusion, suggesting acute disseminated encephalomyelitis versus viral encephalitis versus posterior reversible encephalopathy syndrome (Figure 1). There was also some fluid present in the mastoid air cells bilaterally. Brain MRI with contrast completed three days later revealed only minimal mucosal thickening and fluid in the paranasal sinuses suggestive of acute sinusitis. Neurology was consulted in regard to patient's decreased level of consciousness, ongoing ventilator dependence, and abnormal brain MRI findings.

The patient was suspected to have encephalitis secondary to influenza A and was started on a course of oseltamivir at higher doses of 150 mg twice per day. This regimen was continued for a total of 2 weeks. After completion of the initial course of antibiotics, the patient continued to have persistent fevers. An infectious disease consult was obtained, and a CT of the chest abdomen and pelvis was ordered, showing dense consolidation in the right lower lobe compatible with bronchopneumonia with mucous plugging within the right lower lobe segmental bronchi. These findings suggested a possible bacterial pneumonia superimposed on influenza A infection, and the patient was started on IV piperacillin-tazobactam.

On day 10 of his hospitalization, after nine days of intubation, the patient received a tracheostomy due to failure to extubate and no improvement in mental status. He also received a gastrostomy tube as he was on nasogastric tube feedings since admission due to dysphagia and altered sensorium. Given that the patient remained ventilator-dependent with little improvement in mental status, the patient was transferred to a long-term acute care hospital (LTACH) facility for further specialized care. He did not show any neurologic recovery or improvement in the three months after initial presentation of symptoms. He was transferred to a subacute care facility after the family had a palliative care meeting. Patient was initially a full code but become a do not resuscitate (DNR), as the family felt it best for their loved one at that time. Comfort measures were then instituted. In the fifth month after the initial symptoms, the patient was admitted to an ED, due to episodes of coffee ground emesis (300 mL) from the PEG tube with associated fever and tachycardia (140-150 s). A rectal exam showed no melena. Stool guaiac was negative. A CXR showed multifocal pneumonia in bilateral lobes. A CT of the abdomen showed dilated bowel suggestive of a functional obstruction, i.e., Ogilvie's syndrome, with no evidence of a mechanical obstruction or surgical emergency. He was given a 1-liter bolus of normal saline. He was started on IV pantoprazole and empiric antibiotics (zosyn 3.375 grams, vancomycin 1 gram) for broad coverage. A central line was placed for better venous access, and he was transferred to critical care in a different facility in the same hospital network. In the fifth month after the initial insult, there was no recovery of preinsult neurologic function. Patient was able to open eyes spontaneously on occasion but did not follow verbal command or respond to an external stimuli.

The family had a palliative care meeting to discuss the plan and goals of care. His current neurologic function and his prognosis and quality of life were discussed at length. It was decided by close family members that “compassionate extubation” would be done due to ongoing stress on the patient's body physically and neurologically.

## 3. Discussion

Influenza is an acute viral illness with primary involvement of the respiratory tract. New strains of the influenza virus can spread globally and cause a pandemic to great proportions. The knowledge of influenza's incidence, epidemiology, preventative measures, and vaccinations along with diagnostic tests and therapy are essential to clinical practice in the clinic and hospital medicine [5]. Seasonal and pandemic influenza are the two faces of influenza that affect humans. Seasonal influenza is more prevalent in the fall and winter months. Viral strains are closely monitored, and a yearly updated vaccination is made available to the population, especially at risk populations.

Influenza can be a seemingly harmless viral illness but there are risks of complications especially with at risk populations with comorbidities that equate to an immunocompromised state. At-risk populations include adults 65 years and older, those with chronic illnesses (i.e., heart, lung, and renal), immunocompromised illnesses (i.e., human immunodeficiency virus (HIV), acquired immunodeficiency syndrome (AIDS), leukemias, and lymphomas), medical treatments (i.e., chemotherapy and radiation), and possibly a genetic predisposition commonly seen in Asian/Pacific Islanders [1]. People living in long-term care facilities, pregnant women, and certain racial ethnicities are other populations at risk. Surprisingly, this particular patient did not have typical risk factors that would place him at risk for influenza complications as compared to those who are immunocompromised.

Severe influenza infection represents a leading cause of global morbidity and mortality. Though influenza is considered a viral infection primarily limited to the respiratory system, through clinical reports, there is the suggestion that infection can be associated with clinical syndromes that involve extrapulmonary organ systems [1]. Complications due primarily to influenza include but are not limited to pneumonia, acute respiratory failure, encephalitis, or encephalopathy. In the past, there has been an emergence of a reassorted human-pathogenic influenza A virus strain that has been known to cause increased morbidity and spreads rapidly in the immunologically naïve human population [6, 7]. This patient was found to be positive for influenza A through polymerase chain reaction (PCR) nasal swab discovered on initial lab work completed on admission. He was subsequently started on oseltamivir 75 mg BID that was increased to 150 mg twice per day over the course of a 2-week period given the high suspicion for influenza-associated encephalopathy. In the current literature and research, there is evidence that steroid therapy in the acute phase of IAE has beneficial effects/outcomes on morbidity and mortality rates of patients. He was not started on steroid pulse therapy at the time of diagnosis due to severe diabetes mellitus type II with elevated blood glucose levels and subsequent complications initially on presentation and during the early, immediate disease course.

Symptoms of influenza can include but are not limited to fever, rhinitis, pharyngitis, myalgia, malaise, and headache. For this particular patient, he was having symptoms of coughing, chills, and vomiting and a general feeling of malaise for approximately one week prior to presentation to the ED. He was having symptoms of confusion for approximately one day prior to arrival to the ED. According to the patient's family and fellow coworkers, he had not been feeling like his normal self for a few days prior to presentation, but still decided to continue to go to work. The day he was brought to the ED, his coworkers were present and mentioned that he was having symptoms of worsening nasal congestion and headaches.

Prior case reports has shown that the diagnostic work up in regard to a patient presenting with influenza extrapulmonary signs and symptoms that are neurological-specific includes a CT scan of the brain. Seven cases (39%) had acute abnormalities evident including diffuse edema (5%), focal left parietal and occipital cortical edema (5%), patchy hypodense lesions on the vertex and thalamus with diffuse edema (11%), and superior sagittal thrombosis and cerebral hematomas (5%) [1, 6]. Our patient had an initial CT of the head that showed mucosal thickening throughout the frontal, sphenoid, ethmoid, and maxillary sinuses. Later in the day due to changes in mental status, another CT of the head was completed which showed symmetric areas of decreased attenuation in frontal lobes bilaterally with increased gray-white matter differentiation.

Altered level of consciousness on day two of admission resulted in an order for a CT head without contrast to be ordered in a stepwise fashion. Since there was no change in his level of consciousness in the preceding days, an MRI brain without contrast was completed. This demonstrates the importance of rapid clinical judgment based on patient's status to prevent long-term physical and neurological sequelae.

**Figure 2:**
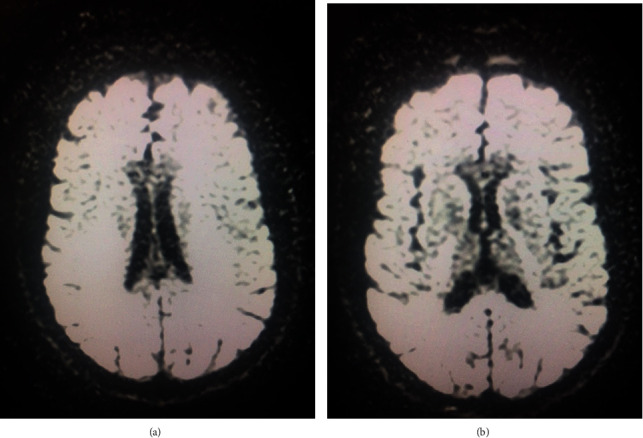
(a) Brain MRI without contrast with diffusion-weighted imaging (DWI). (b) Brain MRI without contrast with apparent diffusion coefficient (ADC).

In another study of ten patients, an MRI scan was completed which showed evidence of abnormalities. Signal abnormalities were seen in the cortex, white matter, and brainstem throughout primarily [1]. Two patient's MRI showed hyperintense signaling on the central splenium of the corpus callosum typical of mild encephalitis/encephalopathy with reversible splenium lesion (MERS) [1]. Three patients showed evidence of vasogenic edema in the frontal cortex in one and in the parietal and occipital cortex in the other two patients. Our patient's MRI showed symmetric high T2 FLAIR signal involving the periventricular white matter and splenium of the corpus callosum with associated restricted diffusion. MRI findings from our patients favored an ongoing inflammatory/encephalopathic process (Figure 2). Lesions of this nature have been called “cytotoxic lesions of the corpus collosum” or “CLOCC” lesions. These lesions of the splenium of the corpus callosum show evidence that cytotoxic edema is a part of the pathophysiology of this disease process. Cytokine activation with subsequent cytotoxic edema is the hypothesis regarding this pathology initiation. The brain MRI without contrast showed the splenial lesion but the last imaging of the head was a CT. It was completed approximately three months later and did not show any evidence of the lesion.

MERS is associated with a milder course and often a better clinical outcome.

Electroencephalography (EEG) testing revealed diffuse slowing typical of encephalitis (79%) and epileptic discharges in (7%). For other patients, their EEG results were deemed within normal limits (7%). Our patient had an EEG that showed irregularity of cerebral function in frontotemporal brain regions.

Lumbar puncture was performed in certain patients (24 of the 28 patients) which demonstrated pleocytosis in 17%, elevated protein levels in 17%, and within normal limits in 46% [1]. PCR of the CSF was positive for influenza in (21%) of the patients who had a lumbar puncture performed [1]. It appears through review of current literature, that PCR-CSF is underutilized in clinical practice; however, it shows some promise to buffet future clinical practice as opposed to utilization in research alone. Our patient had an LP that showed mild lymphocytic pleocytosis which endorses a viral etiology of the encephalitis/encephalopathy. In the literature, a concentration of proinflammatory cytokines can be seen in plasma and CSF samples in those with IAE. An elevation of cytokines, i.e., interleukin (IL)-1b, IL-6, tumor necrosis factor (TNF)-a, and soluble TNF receptor is seen in the plasma and CSF. In our laboratory, we do not have those specific capabilities currently; however, it generates a query if the management would have changed based on the results.

The extrapulmonary manifestations of influenza are broad and vast in nature and include almost every organ system. They are likely the result of host factors (i.e., age, comorbidities, and genetic predisposition), unique viral pathogenesis, or a combination of both entities [1]. Cardiac complications are related to ischemic heart disease and myocarditis. Neurological complications can be profound in nature and vary in presentation including stroke, guillain-barre syndrome, encephalitis/encephalopathy among many others. Acute renal injury and even ocular manifestations have been reported in relation to conjunctivitis that is influenza induced/associated.

Overall, 79% of adult patients with IAE survived with established, evidence-based care [1]. Patients suffered residual neurological defects however which include primarily cognitive deficits and motor weakness in approximately 25% [1]. Those specific symptoms were evident in the immediate three-month period postinsult. Long-term symptoms and sequelae beyond three months are unknown due to under reporting [1]. This patient was transferred to a LTACH facility for a higher level of specialized care. The three-month follow-up shows that this particular patient was nonresponsive. He has occasional episodes of spontaneous eye opening. He was not following verbal command or responding to external stimuli which was also seen in the acute phase of illness. Hypertonicity in all four extremities with significant contractures was noted in the upper extremities and lower extremities. Hypoactive reflexes were also noted on physical examination. CT head in the three-month period after initial insult showed a minimal degree of cerebral and cerebellar cortical atrophy and a minimal degree of central atrophy of the cerebral hemispheres. Decreased attenuation in white matter of both cerebral hemispheres was noted, consistent with demyelination. At that time, no acute findings of hemorrhage or mass effect were seen. At five-month postinsult, the patient did not have any semblance of partial or complete neurologic recovery.

Influenza-associated encephalopathy is a syndrome that mainly manifests as a consciousness disturbance provoked by influenza [8] and is an acute rapidly progressive neurological dysfunction that results chiefly from noninflammatory brain edema. The brain damage is progressive and is without the presence of direct invasion of the virus and inflammatory cells. Adult cases in Europe and the US have been reported since the 2009 pandemic [8].

Influenza can lead to a variety of neurologic complications that are specific clinical entities grouped as IAE. There is also postinfluenza encephalitis, a separate syndrome in and of itself. This patient's case appeared more like encephalopathy that was influenza-associated, in nature.

With acute influenza symptoms such as fever, cough, nasal congestion, and myalgia, symptoms can be lessened with antivirals used less than 48 hours of symptom onset if these specific medications are to be used at all. After 48 hours of symptoms, the use of antivirals is not beneficial or overall recommended to patients. However, neuraminidase inhibitors, i.e., oseltamivir or zanamivir, are commonly used. Usual dosing for oseltamivir is 75 mg twice per day orally or zanamivir 10 mg twice per day by inhalation. Doubling the dose of oseltamivir to 150 mg twice per day has been suggested for critically ill patients and those with a preexisting immunocompromised state of health.

Treatment ranges from a combination of options such as cooling measures to induce a state of mild hypothermia to conserve the body's energy stores. Cooling temperatures can ranged from 32 to 34 degrees Celsius. Lower core body temperatures, i.e., 33 to 36 degrees Celsius, from studies show similar improvements in clinical outcomes. Hypothermia can alleviate excessive cerebral edema while allowing for better protection of the brain from subsequent irreversible neural cell damage and systemic expansion [9].

Lower morbidity and mortality rates and an improved rate of good neurological outcome has been demonstrated in clinical trials. The adamantane, i.e., amantadine, has also been used to prevent and treat influenza A by inhibiting viral particle uncoating and nucleic acid release into the host cell which leads to decreased disease activation in the host. Anticytokine agents, i.e., high-dose methylprednisolone and ulinastatin, have been shown to be effective for severe cases of IAE. As elevated proinflammatory cytokines are seen in the serum and CSF of patients with IAE, treatment modalities that manipulate the cytokine activation and subsequent cytokine storm have promise. Combination therapy modality has shown strong utility in regard to thorough IAE treatment.

This particular patient had symptoms of influenza and a general feeling of malaise. This case illustrates the importance for astute and prompt clinical diagnosis and subsequent treatment of influenza symptoms as its complications can be rapidly progressive. Keen attention and follow-up is of utmost importance to treatment and recovery in the initial phases.
